# CD3+ Tumor-Infiltrating Lymphocytes in Breast Carcinoma: Their Prognostic Role and Association With Clinical Parameters

**DOI:** 10.7759/cureus.75479

**Published:** 2024-12-10

**Authors:** Rishav Chauhan, Vijayalaxmi Patil

**Affiliations:** 1 Pathology, BLDE (Deemed to be University) Shri B M Patil Medical College, Hospital and Research Centre, Vijayapura, IND

**Keywords:** breast carcinoma, cd3, immunohistochemistry, tumor infiltrating lymphocytes, tumor size

## Abstract

Background

Breast carcinoma cases are rising steadily and represent a major cause of mortality and morbidity in India. In response to breast carcinoma, the immune system is activated, resulting in lymphocyte infiltration in and around the tumor nests. This interaction between the tumor and immune system is the basis for studying tumor-infiltrating lymphocytes (TILs). Despite studies on TILs in breast carcinoma, the role of CD3+TILs in predicting patient outcomes remains underexplored. This study aimed to evaluate TILs in breast carcinoma tissues by analyzing CD3 expression through immunohistochemistry (IHC) and examining its association with various prognostic factors, such as histological grade, clinical stage, lymph node status, and hormone receptor status.

Methods

A study was conducted on 45 breast carcinoma cases, documenting various clinicopathological parameters. IHC staining was performed for estrogen receptor (ER), progesterone receptor (PR), human epidermal growth factor (HER2-neu), and CD3 markers on tissue samples. Both intra-tumoral and stromal CD3 TILs were quantified and analyzed in relation to clinicopathological prognostic factors.

Results

In all 45 breast carcinoma cases, intra-tumoral and stromal CD3 expression was assessed. High stromal CD3 expression is documented in the triple-negative breast tumor. CD3 intra-tumoral expression correlated significantly with tumor size (p < 0.035), ER status (p < 0.036), and PR status (p < 0.036). CD3 stromal expression showed no correlation with any of the prognostic parameters.

Conclusion

The current study found a strong correlation between CD3 intra-tumoral expression with tumor size, ER and PR status. The findings imply that CD3 may be a significant factor in breast carcinoma prognosis. There were more stromal CD3 TILs in triple-negative breast carcinoma (TNBC) cases. TILs ought to be examined for their capacity as novel prognostic and predictive indicators, especially in cases of invasive breast cancer, such as triple-negative breast carcinoma.

## Introduction

Breast carcinoma is the most common carcinoma among women globally, accounting for 24% of all carcinoma cases in women. It is also the most prevalent carcinoma among women in India. Breast carcinoma is a multifactorial disease with diverse clinical patterns, pathological features, prognostic variables, and therapy responses [1].

Tumor-infiltrating lymphocytes (TILs) are a mix of T-cells (TIL-Ts), B cells (TIL-Bs), and natural killer (NK) cells that are nested in and around the neoplastic cells. This interaction between the body’s immune system and the carcinoma forms the basis of this study [2]. Most TILs known as stromal TILs (s-TILs) are located in the stromal area immediately adjacent to the tumor. Intra-tumoral TILs (i-TILs) constitute a smaller proportion found inside the tumor. TILs are considered a significant immunological biomarker that, in addition to being detected in other malignancies, represents the immune system’s antitumor response in breast carcinoma [2,3].

Although there is conflicting information on the potential therapeutic benefits of different TIL types, measuring the number of stromal TILs and intra-tumoral TILs may be helpful for prognostic and predictive purposes in breast carcinoma. There may be predictive and prognostic value to the cellular makeup of TILs in breast carcinoma [2-4].

CD3 is present during every stage of T-cell development as it triggers a signaling cascade to activate T-cells following antigen recognition. CD3 is a known T-cell marker. It is essential for antigen identification, T-cell activation, and the subsequent induction of an immunological response specific to the antigen. Presence of TILs indicate a better prognosis in breast carcinoma which further highlights the importance of CD3 as a biomarker. CD3 can have larger impact on breast carcinoma diagnosis and treatment. This study aims to assess the CD3+ subset of TILs, their location, density, and dispersion in breast carcinoma patients and their impact on survival [4,5].

## Materials and methods

Forty-five histologically proven cases of breast carcinoma were included in this study. Their history, clinical stage, tumor grade, lymph node status, estrogen receptor, progesterone receptor and HER2-neu status were recorded. Pre-operatively all these patients had been diagnosed as a case of breast carcinoma. All patients underwent surgery accompanied by axillary lymph node dissection, and none had received pre-operative anti-tumor therapy. Institutional ethical committee approval was obtained for the study in August 2022 from BLDE (DU) Shri B M Patil Medical College, Hospital and Research Center (approval BLDE(DU)/IEC/676/2022-23.)

**Figure 1:**
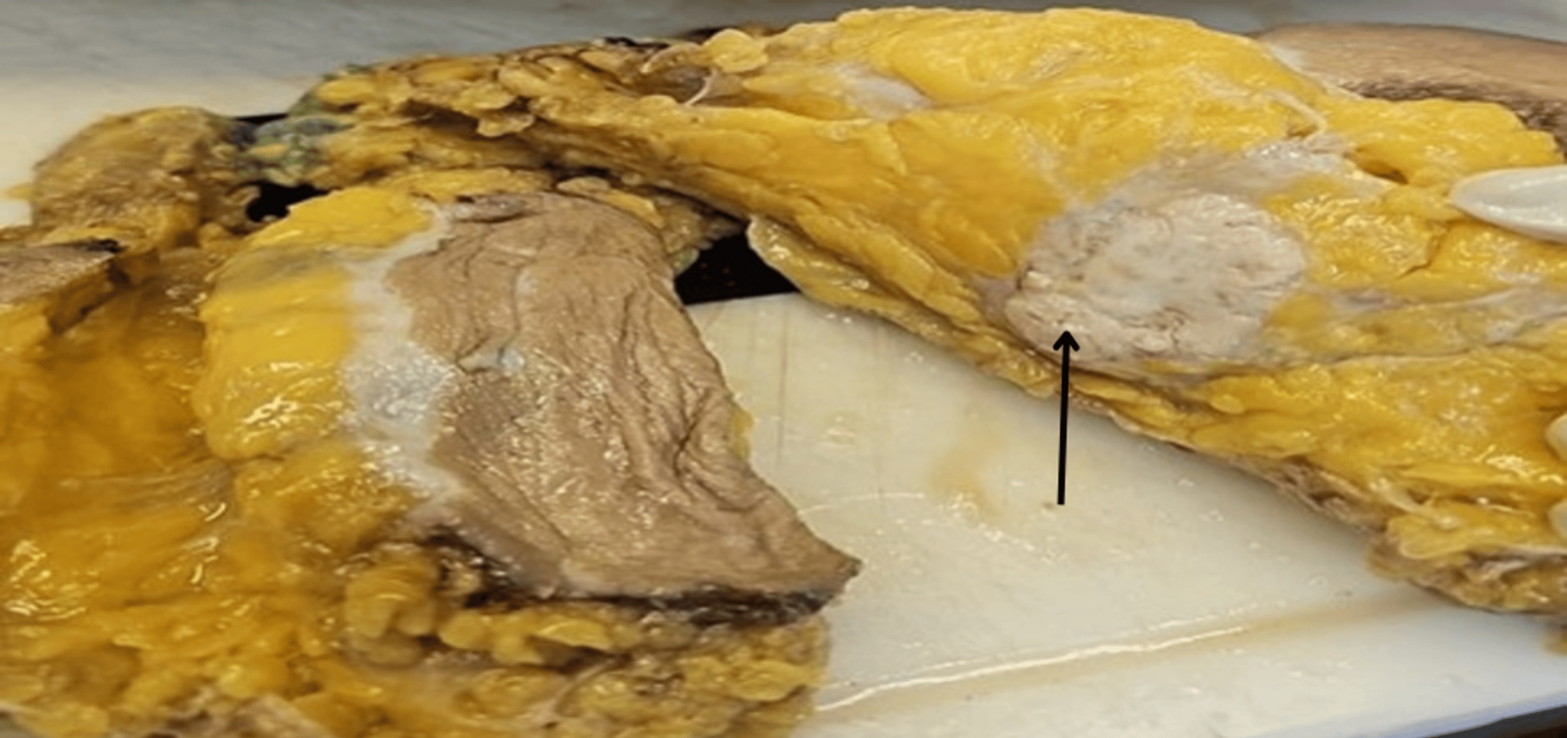
Gross photograph of the cut section of tumor. Arrow showing the white tumor part

All mastectomy specimens were submitted to the histopathology section of the Department of Pathology at Shri B M Patil Medical College, Hospital and Research Center, Vijayapura. The specimen was preserved in 10% formalin and processed routinely. Grossly white color tumor of the invasive breast carcinoma is appreciated (Figure 1). Five sections of 4-micron thickness were prepared from the most suitable tissue block. One section was stained with hematoxylin and eosin for morphological grading according to the Bloom and Richardson grading system of breast cancer. The other four sections were poly-lysine-coated slides and subjected to estrogen receptor (ER)/progesterone receptor (PR), human epidermal growth factor receptor (HER2)-neu and CD3 immunohistochemical staining according to standard protocol.

**Figure 2:**
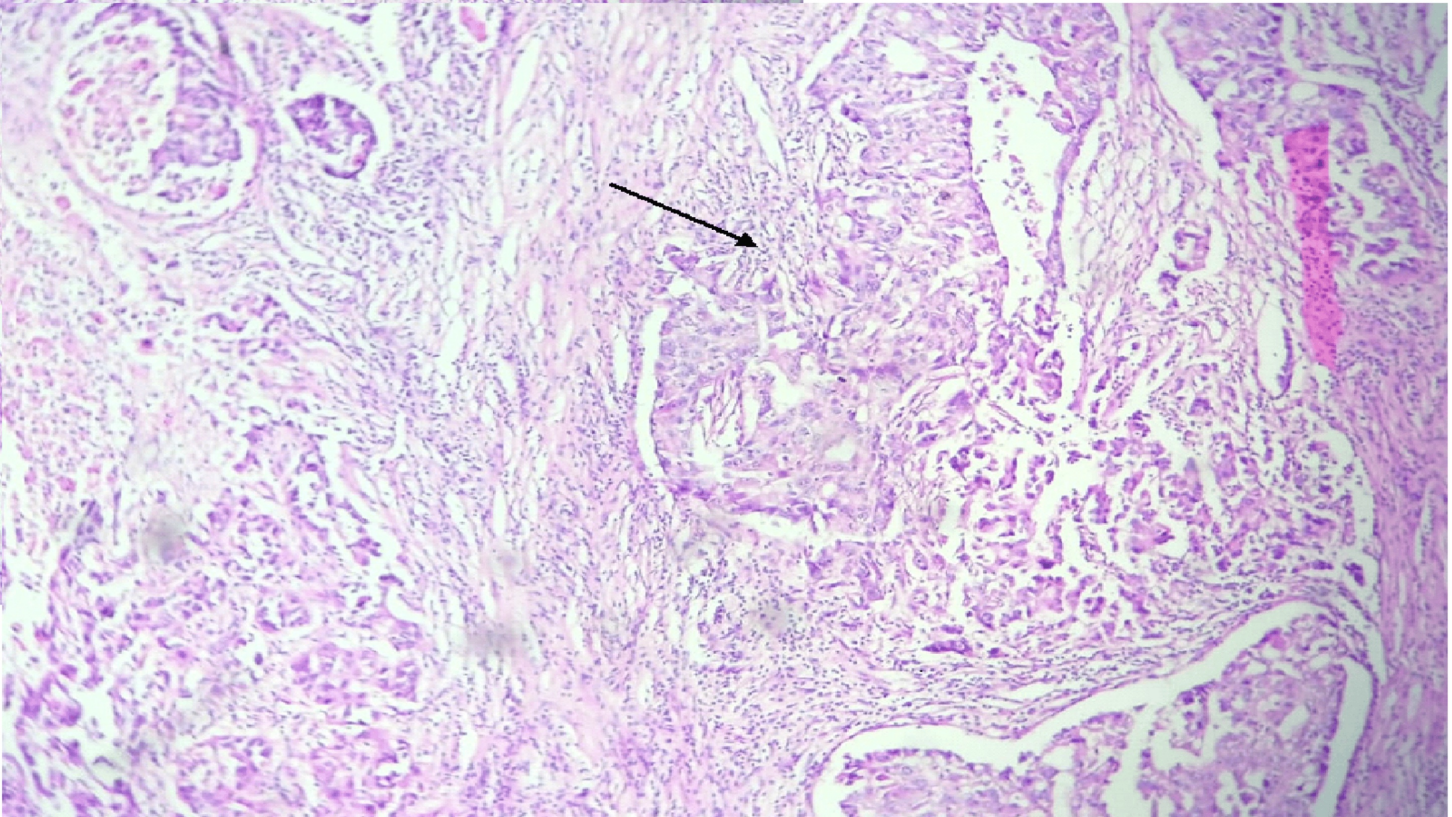
Photomicrograph of invasive breast carcinoma showing stromal tumor infiltrating lymphocytes. (H&E, 100x)

**Figure 3:**
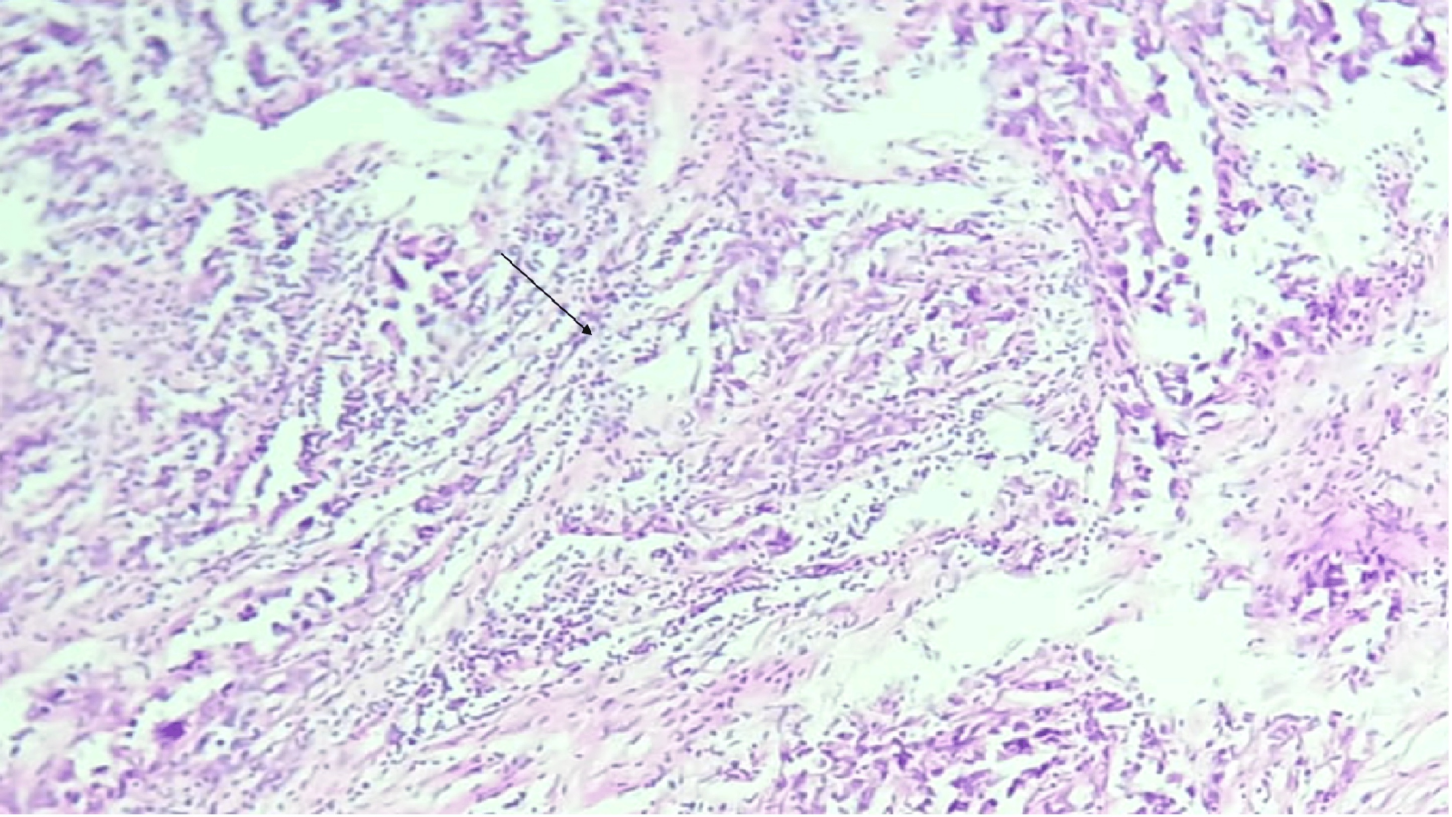
Photomicrograph of invasive breast carcinoma showing intra-tumoral tumor infiltrating lymphocytes (H&E, 100x)

CD3+ TILs were counted in five randomly selected high-power fields at 40x magnification, with the counts then averaged. TIL levels within the tumor and stromal areas were categorized as follows: + (1-25 cells), ++ (26-50 cells), and +++ (51 cells), where a high TIL count was classified as more than 25 positive TILs (++ or +++), and a low TIL count as fewer than 25 positive TILs. Figure of stromal tumor infiltrating lymphocytes (Figure 2) and intra-tumoral infiltrating lymphocytes (Figure 3) has been illustrated. The immunohistochemical (nuclear staining) expression of the markers ER and PR was evaluated based on the Allred scoring system, while HER2 was evaluated based on the extent and intensity of membrane staining. The immunohistochemical expression of CD3 was correlated with prognostic factors such as tumor size, histological grade, lymph node status, ER, PR, and HER2-neu status.

Specimens from patients who had undergone neoadjuvant chemotherapy or radiation therapy were excluded to ensure treatment-naive tissue evaluation. Data was entered into an Excel spreadsheet (Microsoft, Redmond, WA, USA), and descriptive statistics (mean and standard deviation for quantitative data, frequency and proportions for qualitative data) were calculated. To determine the relationship between qualitative variables, inferential statistics such as the chi square test were used. The level of significance was fixed at 5%.

## Results

In this study, cases were categorized into two groups above 40 years and below 40 years of age. There was low CD3 stromal expression in 13 cases (31.1%) and high CD3 stromal expression in 32 cases (68.9%). Among 45 cases, 42 (92.1%) showed low CD3 intra-tumoral expression, while three (7.9%) showed high expression.

In the age demographics of the 38 cases that fell into the above-40 age category, 28 had high CD3 stromal expression (73.7%), and 10 had low CD3 stromal expression (26.3%). Four out of the seven cases in the below-40 age group had high CD3 stromal expression (57.1%), while three cases had low CD3 stromal expression (42.9%). Results of stromal expression of CD3 (Table 1) and intra-tumoral expression of CD3 (Table 2) with different parameters are summarized below.

**Table 1:** Correlation of CD3 stromal expression with various prognostic parameters in breast carcinoma ER: estrogen receptor, PR: progesterone receptor, HER2: human epidermal growth factor

Parameters	Stromal expression of CD3	
	High	Low
Age		
<40 Years	4 (57.1%)	3 (42.9%)
>40 Years	28 (73.7%)	10 (26.3%)
Histologic grade		
I	8 (61.5%)	5 (38.5%)
II	20 (76.9%)	6 (23.1%)
III	4 (66.7%)	2 (33.3%)
Lymph node		
Negative	9 (69.2%)	4 (30.8%)
Positive	23 (71.9%)	9 (28.1%)
Tumor size		
T1	2 (100%)	0 (0%)
T2	22 (71%)	9 (29.0%)
T3	8 (66.7%)	4 (33.3%)
ER status		
Negative	14 (73.7%)	5 (26.3%)
Positive	18 (69.2%)	8 (30.8%)
PR status		
Negative	13 (65%)	7 (35%)
Positive	19 (76%)	6 (24%)
HER2-neu status		
Negative	20 (76.9%)	6 (23.1%)
Positive	12 (63.2%)	7 (36.8%)

**Table 2:** Correlation of CD3 intra-tumoral expression with various prognostic parameters in breast carcinoma ER: estrogen receptor, PR: progesterone receptor, HER2: human epidermal growth factor

Parameters	Intra-tumoral expression of CD3	
	High	Low
Age		
<40 Years	0 (0%)	7 (100%)
>40 Years	3 (7.9%)	35 (92.1%)
Histologic grade		
I	0 (0%)	13 (100%)
II	3 (11.5%)	23 (88.5%)
III	0 (0%)	6 (100%)
Lymph node		
Negative	2 (15.4%)	11 (84.6%)
Positive	1 (3.1%)	31 (96.9%)
Tumor size		
T1	1 (50%)	1 (50%)
T2	1 (3.2%)	30 (96.8%)
T3	1 (8.3%)	11 (91.7%)
ER status		
Negative	3 (15.8%)	16 (84.2%)
Positive	0 (0%)	26 (100%)
PR status		
Negative	3 (15.8%)	16 (84.2%)
Positive	0 (0%)	26 (100%)
HER2-neu status		
Negative	1 (3.8%)	25 (95.2%)
Positive	2 (10.5%)	17 (89.5%)

The cases of patients over 40 were examined for CD3 intra-tumoral expression, out of which three showed high CD3 intra-tumoral expression, while 35 showed low CD3 expression. Seven cases under 40 years had low CD3 expression, accounting for all cases in the particular age category of patients. No statistically significant data showed a correlation between CD3 expression and population age, as indicated by the p values of stromal and intra-tumoral expression, which are 0.375 and 0.442, respectively.

Of the 45 cases examined in this study, 32 cases had lymph node involvement. Of these 32 patients, 23 (71.9%) had high CD3 stromal expression, while nine (28.1%) had low CD3 stromal expression. One case (3.1%) had high intra-tumoral CD3 expression, while 31 cases (96.9%) had low intra-tumoral CD3 expression. Thirteen cases in the current investigation showed no involvement of lymph nodes. Nine cases (69.2%) had high CD3 stromal expression, while four cases (30.8%) had low CD3 stromal expression. Two patients (15.4%) had high CD3 intra-tumoral expression, while 11 cases (84.6%) had low CD3 intra-tumoral expression. No statistically significant correlation was seen in between CD3 expression and lymph node involvement, as evidenced by the p values of stromal and intra-tumoral CD3 expression, which are 0.859 and 0.135, respectively.

Eight of the 13 grade I carcinoma cases (61.5%) had high CD3 stromal expression, while six cases (38.5%) had low CD3 stromal expression. Twenty of the 26 grade II cases (76.9%) had high CD3 stromal expression, while the remaining six cases (23.1%) had low CD3 stromal expression. Four of the six Grade III cases (66.7%) had high CD3 stromal expression; the other two had low CD3 stromal expression (33.3%). Grade I carcinoma cases had low intra-tumoral expression of CD3, which accounted for all 13 grade I cases. Of the 26 grade II cases, three (11.5%) had high CD3 intra-tumoral expression, while 23 (88.5%) had low CD3 intra-tumoral expression. Low CD3 intra-tumoral expression was present in six Grade III patients, accounting for 100% of the cases. The relationship between CD3 expression and histological grade was statistically insignificant, as seen by the p values of stromal and intra-tumoral CD3 expression, which were 0.568 and 0.309.

High CD3 stromal expression was observed in two T1 cases. In 31 cases of tumor stage T2, 22 (71%) cases exhibited high CD3 stromal expression, while nine (29%) had low CD3 stromal expression. Four of the 12 tumor stage T3 patients (33.3%) had low CD3 stromal expression, while eight of the 12 cases (66.7%) had high CD3 stromal expression. CD3 intra-tumoral expression was seen in two tumor-stage T1 patients, 50% of T1 cases had high intra-tumoral expression and 50% of T1 cases had low intra-tumoral expression. 30 cases (96.8%) showed low CD3 intra-tumoral expression, whereas one case (3.2%) of the 31 tumor stage T2 cases had high CD3 intra-tumoral expression. There was low CD3 intra-tumoral expression in 11 cases (91.7%) and high CD3 intra-tumoral expression in one case (8.3%) when the tumor reached stage T3. The p-value of 0.629 for stromal CD3 expression was not statistically significant as no correlation between CD3 expression and tumor was noted. The intra-tumoral CD3 expression and tumor size p-value of 0.035 indicated a significant association between the two variables.

Of the 26 ER-positive patients, 18 cases (69.2%) showed high stromal CD3 expression, while eight cases (30.8%) showed low expression. In all 26 ER-positive cases, low intra-tumoral CD3 expression was seen (100%). Fourteen (73.7%) of the 19 patients with ER-negative expression had high CD3 stromal expression, while five (26.3%) had low CD3 stromal expression. Three instances (15.8%) had high CD3 intra-tumoral expression, while 16 cases (84.2%) had low CD3 intra-tumoral expression. A statistically insignificant correlation was found between stromal CD3 expression and ER, as indicated by the p-value of 0.745 for stromal CD3 expression. A statistically significant correlation was observed between intra-tumoral CD3 expression and ER, with a p-value of 0.036.

Out of 25 PR-positive cases, high stromal CD3 expression was noted in 19 cases (76%), and low stromal CD3 expression was seen in six cases (24%). PR-positive low intra-tumoral CD3 expression was noted in all 26 cases (100%). Among 20 cases with PR-negative expression, high CD3 stromal expression was noted in 13 cases (65%) and low CD3 stromal expression in seven cases (35%). High CD3 intra-tumoral expression was noted in three cases (15.8%), and low CD3 intra-tumoral expression was noted in 16 cases (84.2%). The p-value of stromal CD3 expression was 0.419, showing a statistically insignificant association between stromal CD3 expression and PR. The p-value of intra-tumoral CD3 expression was 0.036, showing a statistically significant association between intra-tumoral CD3 expression and PR.

**Figure 4:**
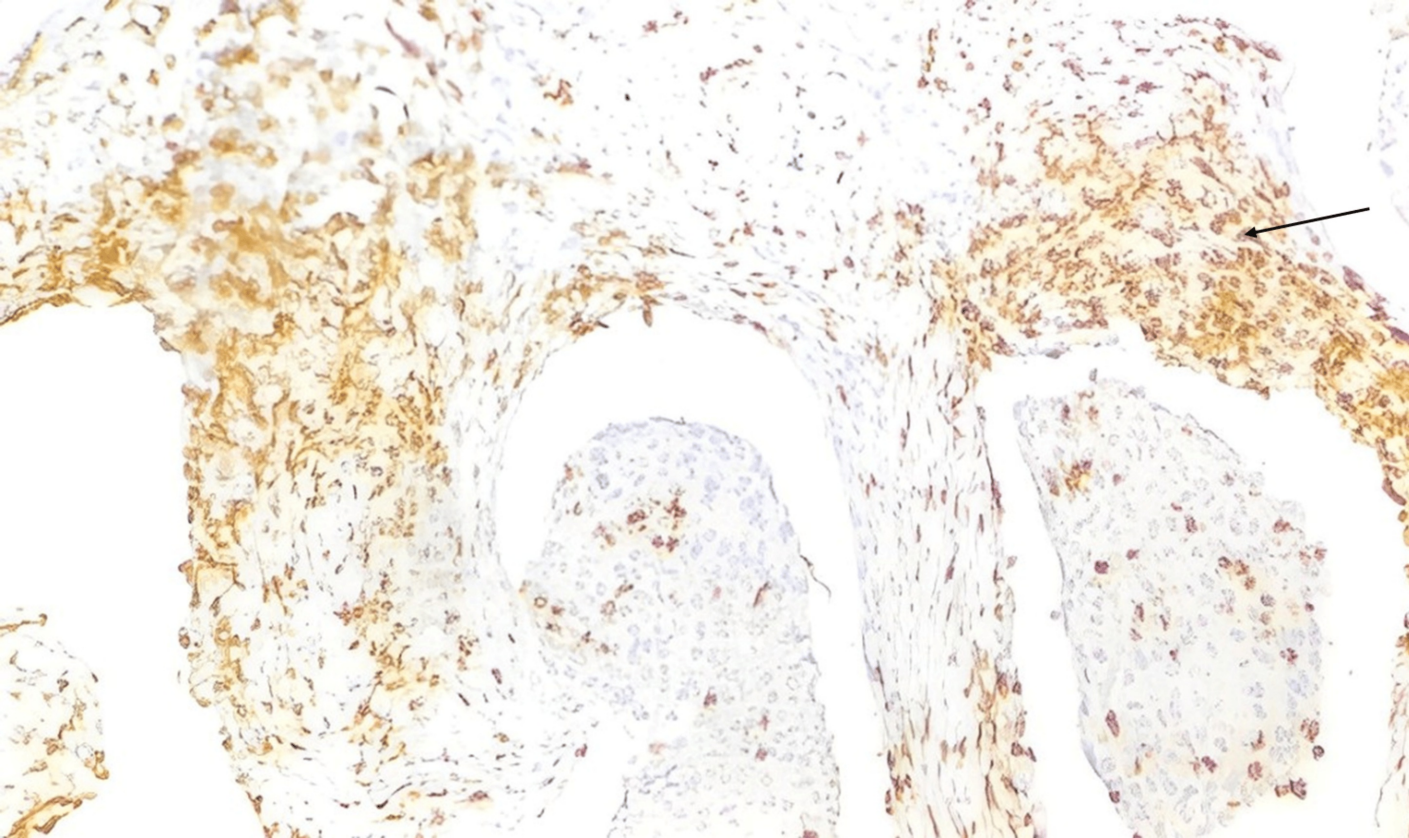
Photomicrograph of high stromal CD3 positivity in invasive breast carcinoma (20x view)

Out of 19 HER2-neu positive cases, high stromal CD3 expression was noted in 12 cases (63.2%), and low stromal CD3 expression was seen in seven cases (36.8%). HER2-neu positive high intra-tumoral CD3 expression was noted in two cases (10.5%), and low intra-tumoral CD3 expression was noted in 17 cases (89.5%). Among 26 cases with HER2-neu negative expression, high CD3 stromal expression was noted in 20 cases (76.9%) and low CD3 stromal expression in six cases (23.1%). High CD3 intra-tumoral expression was noted in one case (3.8%), and low CD3 intra-tumoral expression in 25 cases (95.2%). The p-value for stromal CD3 expression was 0.314, and intra-tumoral CD3 expression was 0.375, showing a statistically insignificant association between CD3 expression and HER2-neu. Below is an illustrated figure of immunohistochemistry of high stromal CD3 positivity in invasive breast carcinoma (Figure 4).

## Discussion

Prognostic parameters of breast carcinoma include age, modified Scar Bloom Richardson grade, tumor size, lymph node involvement, ER, PR, and HER2-neu status of the patient. Advancement of new therapeutic modalities and progress in molecular diagnostics have increased interest in novel prognostic and predictive tools [5].

The presence of tumor-infiltrating lymphocytes in the cancer tissue indicates their role in the immune response in the control and elimination of tumor cells. Higher counts of TILs are associated with a favorable prognosis [6]. The role of tumor-infiltrating lymphocytes in invasive breast carcinoma and its correlation with different prognostic variables are a matter of discussion. The location of TILs, whether they are stromal or intra-tumoral is also important. TILs are now considered a hallmark of cancer development [7].

In this study, we analyzed 45 cases of invasive breast carcinoma and found a correlation between the CD3 marker's intra-tumoral and stromal expression and the patient's age. The average age was 54 years, ranging from 33 to 82 years. Analysis revealed p < 0.375 and p < 0.442. There was no statistically significant correlation between CD3 expression and age. These results are consistent with findings by Rathore et al., who similarly observed no age-related correlation with CD3 expression in breast carcinoma across 150 cases [7].

Of the 45 patients in this study, there was no significant connection between stromal and intra-tumoral CD3 expressions and lymph node involvement (p < 0.859 and p < 0.135, respectively). Our finding was in contrast with the study done by Rathore et al., who examined 150 cases of invasive breast carcinoma and reported significant association between lymph node involvement and CD3 expression [7].

In this study, among the 45 cases of invasive breast carcinoma, no significant correlation was found between CD3 expression, whether stromal or intra-tumoral, and histologic grade as indicated by p < 0.568 and p < 0.309. This finding was consistent with the study done by Locy et al., who looked at 37 cases of invasive breast carcinoma and found no significant relationship between CD3 expression and histologic grade [8].

In this study, tumor sizes ranged from 2 to 12 cm and were categorized as T1 for tumors under 2 cm, T2 for those between 2 and 5 cm, and T3 for tumors exceeding 5 cm. A p < 0.629 indicated no significant association between stromal CD3 expression and tumor size, aligning with the study of Balkenhol et al. of 94 invasive breast carcinoma cases, which also found no substantial link between tumor size and CD3 expression [9]. In contrast, the p < 0.035 for intra-tumoral CD3 expression and tumor size, showing a significant correlation, similar to findings by Konig et al., who identified a strong association in a study of 87 breast carcinoma cases [10].

In this study, the ER status was also analyzed across 45 cases. The p-value for stromal CD3 expression and ER status was 0.745 showing no significant correlation. The p < 0.036 for intra-tumoral CD3 expression and ER status, demonstrating a significant association similar to the results reported by Konig et al [10]. For PR, the stromal CD3 expression showed a p < 0.419, indicating no significant relationship. The p < 0.036 for intra-tumoral CD3 expression with PR status, revealing a significant association, in line with Konig et al., study [10]. In terms of HER2-neu, p < 0.314 and p < 0.375 for stromal and intra-tumoral CD3 expressions respectively, showing no significant correlation. This aligns with Konig et al. who also found no significant association in a study of 87 cases [10].

In the 10 triple-negative breast carcinoma (TNBC) cases, seven exhibited high stromal CD3 expression, and one showed significant intra-tumoral expression. TNBC, which lacks HER2, PR, and ER, has a poorer prognosis due to its aggressive nature [11]. Previous studies, such as those by Divyapriya et al., and Vaid et al., observed higher CD3 expression in the stroma for TNBC [12,13]. Rapoport et al. noted that high TIL levels in TNBC correlated with prolonged survival, while HER2-positive patients showed no such correlation [14]. Koletsa et al. reported that higher stromal TIL density was significantly correlated with higher tumor grade, proliferation and TNBC cases [15].

On follow-up of the 45 cases, 12 patients are doing well and have no significant health issues. Five cases are receiving hormone replacement therapy, and three cases have received chemotherapy after surgery. Five deaths were documented within two years of surgery among the study subjects. Efforts are being made to follow up with the remaining cases.

Limitations of the study

More TNBC cases are needed for a better evaluation and correlation in future studies.

Future directions

A study with a larger sample size is needed, as intra-tumoral CD3 expression positively correlated with tumor size, ER, and PR but showed no correlation with age, histologic grade, lymph node status, and HER2-neu. Further evaluation of CD3+ TILs is needed as it can give new options in breast carcinoma diagnosis.

## Conclusions

This study found a significant correlation between CD3 intra-tumoral expression and tumor size, ER, and PR status. These findings suggest that CD3+ TILs may play a significant role in breast carcinoma prognosis. Triple-negative breast carcinomas showed a higher presence of stromal CD3 TILs. Regular assessment of tumor-infiltrating lymphocytes is recommended for their potential as new prognostic and predictive markers, particularly in aggressive breast cancer cases like triple-negative types. In breast carcinoma, CD3+ TILs may serve as an independent marker associated with a favorable prognosis.

## References

[1] Sun XY, Wang CQ, Mao Y, Zhang ZQ, Cui J, Dong XN, Wang HB (2024). Prognostic value and distribution pattern of tumor infiltrating lymphocytes and their subsets in distant metastases of advanced breast cancer. Clin Breast Cancer.

[2] Karki S, Tiwari SB, Basnet A (2022). Evaluation of tumor infiltrating lymphocytes in breast carcinomas. J Pathol Nepal.

[3] Volm MD, Shapiro RL, Demaria S (2001). Development of tumor-infiltrating lymphocytes in breast cancer after neoadjuvant paclitaxel chemotherapy. Clin Cancer Res.

[4] (2021). Diagnostic Immunohistochemistry. 5th ed. https://shop.elsevier.com/books/diagnostic-immunohistochemistry/dabbs/978-0-323-72172-1.

[5] Cabuk FK, Aktepe F, Kapucuoglu FN, Coban I, Sarsenov D, Ozmen V (2018). Interobserver reproducibility of tumor-infiltrating lymphocyte evaluations in breast cancer. Indian J Pathol Microbiol.

[6] Al-Saleh K, Abd El-Aziz N, Ali A (2017). Predictive and prognostic significance of CD8(+) tumor-infiltrating lymphocytes in patients with luminal B/HER 2 negative breast cancer treated with neoadjuvant chemotherapy. Oncol Lett.

[7] Rathore AS, Kumar S, Konwar R, Srivastava AN, Makker A, Goel MM (2013). Presence of CD3+ tumor infiltrating lymphocytes is significantly associated with good prognosis in infiltrating ductal carcinoma of breast. Indian J Cancer.

[8] Locy H, Verhulst S, Cools W (2022). Assessing tumor-infiltrating lymphocytes in breast cancer: a proposal for combining immunohistochemistry and gene expression analysis to refine scoring. Front Immunol.

[9] Balkenhol MC, Ciompi F, Świderska-Chadaj Ż (2021). Optimized tumour infiltrating lymphocyte assessment for triple negative breast cancer prognostics. Breast.

[10] König L, Mairinger FD, Hoffmann O (2019). Dissimilar patterns of tumor-infiltrating immune cells at the invasive tumor front and tumor center are associated with response to neoadjuvant chemotherapy in primary breast cancer. BMC Cancer.

[11] Yazaki S, Shimoi T, Yoshida M (2023). Integrative prognostic analysis of tumor-infiltrating lymphocytes, CD8, CD20, programmed cell death-ligand 1, and tertiary lymphoid structures in patients with early-stage triple-negative breast cancer who did not receive adjuvant chemotherapy. Breast Cancer Res Treat.

[12] Divyapriya C, Kannan A, Raghavan V (2021). Expression of CD4, CD8 biomarkers in invasive carcinoma of breast with clinicopathological correlation. J Pharm Res Int.

[13] Vaid PM, Puntambekar AK, Jumle NS (2022). Evaluation of tumor-infiltrating lymphocytes (TILs) in molecular subtypes of an Indian cohort of breast cancer patients. Diagn Pathol.

[14] Rapoport BL, Nayler S, Mlecnik B (2022). Tumor-infiltrating lymphocytes (TILs) in early breast cancer patients: high CD3(+), CD8(+), and immunoscore are associated with a pathological complete response. Cancers (Basel).

[15] Koletsa T, Kotoula V, Koliou GA (2020). Prognostic impact of stromal and intratumoral CD3, CD8 and FOXP3 in adjuvantly treated breast cancer: do they add information over stromal tumor-infiltrating lymphocyte density?. Cancer Immunol Immunother.

